# Genetic and Behavioral Predictors of Long-Term Weight Loss Maintenance: A Systematic Review of Evidence From Observational and Genetic Studies

**DOI:** 10.7759/cureus.88571

**Published:** 2025-07-23

**Authors:** Kingsley O Ozojide, Esther M Adjei, Oluwafolakemi M Aderinola, Okelue E Okobi, Chiazor B Nguma

**Affiliations:** 1 Public Health, Nottingham Trent University, Nottingham, GBR; 2 Internal Medicine, The Trust Hospital Company Limited, Accra, GHA; 3 Family Medicine, Larkin Community Hospital Palm Springs Campus, Miami, USA; 4 Family Medicine, IMG Research Academy &amp; Consulting LLC, Homestead, USA; 5 General Medicine, University of Port-Harcourt College of Health Sciences, Port-Harcourt, NGA

**Keywords:** behavioral predictors, fto, gene-behavior interaction, obesity, polygenic risk, precision health, weight maintenance

## Abstract

Background: Long-term weight loss maintenance remains a significant challenge in obesity management, despite advances in behavioral, dietary, and medical interventions. The objective of this study is to identify consistent genetic and behavioral predictors associated with sustained weight loss in adults with overweight or obesity.

Methods: We searched PubMed, Embase, PsycINFO, Web of Science, and Google Scholar for peer-reviewed studies published from January 2010 to April 2025. Eligible studies included observational and genetic investigations involving adults who maintained at least 10% weight loss for one year or more. Quality was assessed using the Newcastle-Ottawa Scale (NOS) and genetic validity criteria. A thematic synthesis categorized predictors as behavioral or genetic.

Results: 24 studies met the inclusion criteria (15 observational, nine genetic). Consistent behavioral predictors included increased physical activity, dietary restraint, low disinhibition, and improved psychological health. Genetically, FTO risk alleles and higher polygenic risk scores were associated with weight regain; however, structured behavioral interventions mitigated this effect. Specific gene variants (e.g., PPARγ, TIMP4) were linked to enhanced weight loss outcomes in response to multidisciplinary interventions.

Conclusion: Both genetic and behavioral factors independently and interactively influence long-term weight loss maintenance. Integrating genetic risk profiling with personalized behavioral strategies may improve obesity treatment outcomes.

## Introduction and background

To maintain long-term weight loss, an individual must keep at least 10% of their initial weight reduction off for at least one year. Regardless of the several advances in obesity treatment alongside the availability of diverse weight-loss programs, keeping the weight off is still one of the biggest challenges in managing obesity, possibly as a result of metabolic adaptations, difficulties in sustaining behavioral changes (long term), and psychological factors [1]. Most interventions, including dieting, exercising more, using drugs, or surgery, allow individuals to slim down, but very few manage to keep the extra weight off for a long time [2,3]. Most individuals are likely to get back 80% of their lost excess weight within one to five years, showing that obesity is a hard issue to solve [4]. Sustaining a decrease in body weight is important for public health, as approximately 5% to 10% reduction in body weight may significantly lower the risk of heart disease by approximately 15% to 20%, type 2 diabetes by more than 50%, and certain obesity-related cancers, while also improving mental and overall health [4-8]. This means that researchers need to identify the factors that help some obese people stay healthy and lose weight while others do not [6]. It has often been the case that research in this field mainly looked at factors like what people eat, how much they exercise, their willingness to view their actions, and the support network they have around them [7,8]. While some behavioral strategies are very helpful, the mixed results suggest that biology may play an additional role in how people keep their weight stable [9]. In the last few years, genetic studies have given us new knowledge about how genes work with lifestyle habits to influence our weight over many years [10]. Some people are more likely to gain weight due to genes, which influence appetite control, how much they eat, energy burning, and how they respond psychologically to eating healthy and exercising [11,12].

Despite growing interest in this field, there is lack of comprehensive studies that integrate findings from both observational behavioral studies and genetic investigations to identify consistent predictors of successful long-term weight loss maintenance [13]. Combining the behavioral and genetic predictors can lead to more personalized and effective weight management strategies [14]. For this reason, the objective of this systematic review is to understand and bring together information from studies that explore the factors linked to long-term weight loss maintenance in adults. The main objective of the study is to systematically review existing literature on genetic and behavioral predictors of successful long-term weight loss maintenance in adults.

## Review

Materials and methods

Eligibility Criteria and Search Strategies

The review was done as per the guidelines of the Preferred Reporting Items for Systematic Reviews and Meta-Analyses (PRISMA). The research question was built according to the PICO (population, intervention, comparison, outcome) format, focusing on adults (18+) with overweight or obesity (population) and assessing aspects related to genetics and habits (intervention). It did not include a comparison group (comparison) and aimed to explore ways to maintain weight loss (outcome). This review set the standard for long-term weight loss maintenance, as continuing to hold on to a minimum 10% loss from the start for a year or longer. We analyzed studies that looked at either genetics or behavioral traits, or both, to see if they played a part in successful or unsuccessful weight loss maintenance.

Studies were identified after searching PubMed, Embase, PsycINFO, Web of Science, and Google Scholar that were published from January 1, 2010, to April 1, 2025. Only studies that have gone through the peer-review process and are written in English were analyzed. Searching involved adding pertinent MeSH terms and keywords and joining them with Boolean operators. The search strategy and the different databases used are displayed in Table 1. We also checked the references in accepted papers and recent systematic reviews to find other studies.

**Table 1:** Search strategy overview

Category	Details
Databases Searched	PubMed, Scopus, Web of Science, Google Scholar
Time Frame	January 2010 to April 2025
Language	English only
Search Terms	#1 AND #2 AND #3
#1 (Population)	“Obesity” OR “overweight” OR “weight loss” OR “weight maintenance”
#2 (Predictors)	“genetic predictors” OR “gene variants” OR “SNPs” OR “behavioral factors” OR “dietary behavior” OR “physical activity” OR “psychological traits”
#3 (Outcome)	“long-term weight loss maintenance” OR “weight regain” OR “successful weight maintenance”

Inclusion Criteria

Studies were eligible if they were original, peer-reviewed articles written in English, involving adults over 18 years old with overweight or obesity. Only manuscripts that looked at genetics or behaviors connected to long-term low weights were eligible. Cohort, case-control, cross-sectional, and genetic association studies were included in this review.

Exclusion Criteria

Reviews, meta-analyses, editorials, letters to the editor, conference abstracts, and dissertations without complete and peer-reviewed PubMed articles were not used. Studies were excluded if they did not analyze behavioral or genetic predictors. Research done with non-human cells or in the laboratory, along with papers not written in English, was excluded.

Screening Process

All search results were imported into the EndNote software to filter out any duplicates. Two reviewers looked at the titles and abstracts to see if they met the necessary criteria. All the articles were checked in their entirety to ensure final qualification. Any differences between reviewers were resolved either through consensus or consulting a third reviewer.

Quality Assessment

To ensure methodological rigor, the quality of included studies was assessed using appropriate validated tools based on study design. Observational studies were evaluated using the Newcastle-Ottawa Scale (NOS), which considers selection, comparability, and outcome/exposure assessment [15]. Randomized controlled trials (RCTs) were appraised using the Critical Appraisal Skills Programme (CASP) Randomised Controlled Trial Checklist, focusing on randomization, blinding, and outcome reporting [16]. For non-randomized intervention studies, the Risk of Bias in Non-randomized Studies of Interventions-I (ROBINS-I) tool was applied to assess bias across seven key domains [17]. Systematic reviews and meta-analyses were evaluated using the Revised Assessment of Multiple Systematic Reviews (R-AMSTAR), which scores eleven domains related to review methodology and transparency [18]. 

Data Extraction

Extracted data contained the study title, information about the first author, when the study was published, the country it was carried out in, the study design, sample size, description of participants, what was measured, how long subjects maintained their weight, and main findings. Data was separately extracted and compared by each of the two reviewers to validate it.

Data Analysis

A thematic synthesis approach was used to analyze and interpret the findings of the included studies. Studies were grouped based on categories of predictors, namely, genetic and behavioral factors that influence long-term weight loss maintenance. Given the heterogeneity of study designs and outcomes, no statistical pooling or meta-analysis was conducted. Additionally, most included studies did not report statistical tests such as effect sizes, odds ratios, or confidence intervals in a consistent manner; therefore, these were not analyzed quantitatively. Instead, recurring predictive factors were identified and summarized narratively to highlight consistent trends and areas requiring further investigation.

Results

A total of 320 studies were found through the search of online databases. Once 72 duplications were removed, we read the titles and abstracts of 248 studies and excluded 170 articles. Among the 78 full-text articles looked at, 54 were removed because they either had unsuitable results, had no relevance to our topic, or were not suitable types of articles (like reviews or editorials). Out of the original number, only 24 studies were left for the final analysis. According to location, nine (37.5%) of the studies were from North America, six (25%) were from Europe, five (20.8%) were from Asia, and four (16.7%) covered other, different regions. There were 15 observational studies and nine genetic association studies used in the analysis.

**Figure 1:**
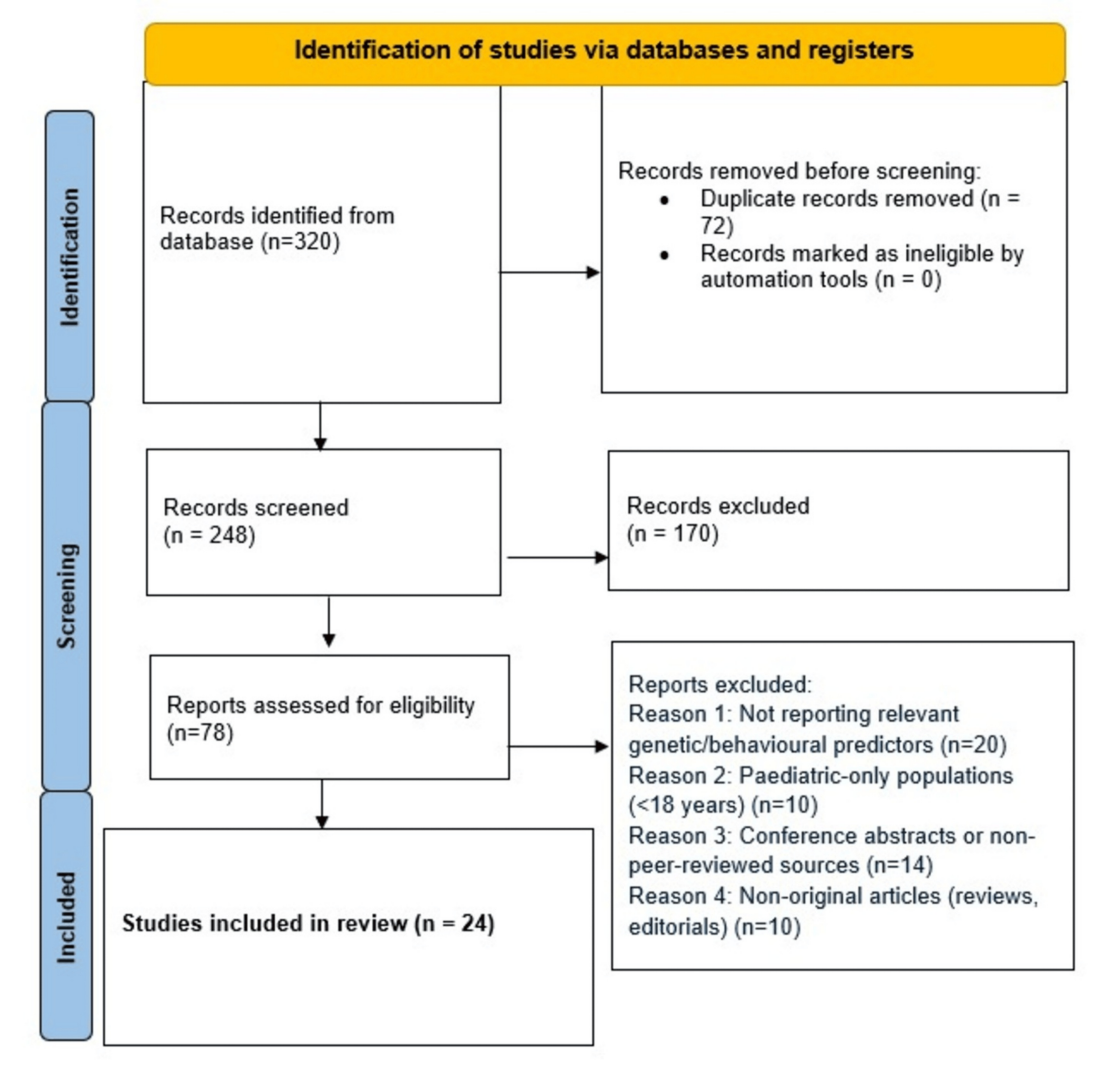
PRISMA flow diagram indicating the study selection and inclusion process n: Number of studies; PRISMA: Preferred Reporting Items for Systematic Reviews and Meta-Analyses

Thematic analysis grouped the information found under behavior and genetics as predictors of long-term weight loss maintenance. People who did well tended to be physically active, restrain their diet, watch how they eat, and score low on binge eating or lack of restraint. Those with good self-esteem and low depression likelihood had better results. According to genetics, some risk alleles, primarily in the FTO gene, were linked to gaining back weight, though usually the effects were reduced by healthy habits. All things considered, using both types of models at the same time resulted in better predictions about weight management, highlighting the potential of customized techniques for managing weight. Figure 1 shows the PRISMA flow diagram outlining the study selection process for the systematic review and meta-analysis.

Table 2 summarizes all the studies included in this review, detailing the reference/citation, study design, study population, and key findings.

**Table 2:** Summary of included studies: study design, population, and key findings NR: Sample size not reported in the source publication; RCT: Randomized controlled trial; PCOS: Polycystic ovary syndrome; CBT: Cognitive behavioral therapy; SNP: Single nucleotide polymorphism

Reference/citation	Study Design	Study Population	Sample Size	Key Findings
Sawamoto et al. [1]	Prospective cohort	Adults with obesity	86	Self-regulation and social support predicted long-term weight loss
Xiang et al. [2]	Systematic review & meta-analysis	Various adult populations	6,951	FTO genotype has minimal impact on weight loss from interventions
Weiland et al. [3]	Observational cohort	Children/adolescents with obesity	NR	Parental support and baseline BMI predicted weight loss
Chen et al. [4]	Pilot study	Adults with obesity	34	Neural and genetic markers associated with treatment outcomes
Papandonatos et al. [5]	RCT	Lifestyle intervention participants	3,940	Genetic predisposition influenced weight loss and regain
Gupta et al. [6]	Systematic review	Adults’ post-bariatric surgery	24	Genetics may predict weight loss outcomes after surgery
Paixão et al. [7]	Systematic review	Weight loss registry participants	NR	Maintenance linked to physical activity and self-monitoring
Varkevisser et al. [8]	Systematic review	Adults in weight loss programs	NR	Motivation and psychological traits are key determinants
Sorgente et al. [9]	Systematic review of reviews	Overweight and obese adults	NR	Web-based interventions support weight loss and maintenance
Chopra et al. [10]	Systematic review	Adults in lifestyle interventions	NR	Self-efficacy and adherence predict outcomes
Agnew et al. [11]	Systematic review	Women with endometrial cancer	NR	Weight reduction improves survival outcomes
Montesi et al. [12]	Review article	Adults with obesity	NR	Multidisciplinary approaches support long-term weight loss maintenance
Lamiquiz-Moneo et al. [13]	Observational study	Overweight and obese adults	788	Genetic variants may influence weight loss responsiveness
Mancini et al. [14]	Systematic review	Adults with obesity	322	Mediterranean diet supports long-term weight loss
Aller et al. [19]	Intervention study	Severely obese adults	587	Genetic predictors influence success in multidisciplinary programs
Hellberg et al. [20]	Observational study	Women with PCOS	55	Weight changes linked to adipose tissue genes
González-Herrera et al. [21]	Cross-sectional	Mayan school-aged children	621	FTO variants linked to obesity/overweight in children
de Luis et al. [22]	Intervention study	Postmenopausal obese females	111	PERILIPIN gene variant predicts weight loss after diet
Rigamonti et al. [23]	Intervention study	Obese adolescents	45	DNA methylation of clock genes linked to short-term weight loss
Aurich et al. [24]	Experimental	Adults undergoing lifestyle change	NR	DNA methylation impacted by weight loss lifestyle
van Dijk et al. [25]	Review	General obese population	NR	Epigenetic mechanisms are involved in obesity development
Palavras et al. [26]	RCT	Adults with binge eating and high BMI	98	CBT plus weight loss program improves outcomes
Coppedè et al. [27]	Observational study	Obese individuals’ post-bariatric surgery	45	Appetite gene methylation predicts weight loss
Vitolo et al. [28]	Observational study	Severely obese individuals	100	SNP in ghrelin gene predicts weight loss after surgery

The methodological quality of all included systematic reviews and meta-analyses was assessed using the R-AMSTAR tool. This instrument evaluates 11 key domains of systematic review quality, including protocol availability, literature search strategy, duplicate data processes, assessment and incorporation of study quality, publication bias evaluation, and conflict of interest reporting. Each domain is scored from 1 (lowest) to 4 (highest), resulting in a total possible score ranging from 11 to 44. The individual domain scores and total quality scores for each study are summarized in Table 3.

**Table 3:** Quality assessment of systematic reviews and meta-analyses using the R-AMSTAR tool R-AMSTAR domains include: 1: A priori design; 2: Duplicate study selection and data extraction; 3: Comprehensive literature search; 4: Inclusion of grey literature; 5: Listing of included and excluded studies; 6: Characteristics of included studies; 7: Quality assessment of included studies; 8: Appropriate use of quality assessments in conclusions; 9: Appropriate synthesis methods; 10: Assessment of publication bias; 11: Conflict of interest disclosure Scores range from 1 (low quality) to 4 (high quality) per domain; maximum total score = 44 R-AMSTAR: Revised Assessment of Multiple Systematic Reviews

Study	1	2	3	4	5	6	7	8	9	10	11	Score
Xiang et al. [2]	4	4	4	2	3	4	4	4	4	2	4	39
Weiland et al. [3]	3	3	4	1	3	4	3	3	3	1	3	31
Gupta et al. [6]	4	4	4	2	4	4	4	4	3	2	4	39
Paixão et al. [7]	4	4	3	3	3	4	4	4	4	3	4	40
Varkevisser et al. [8]	4	4	4	3	3	4	4	4	3	2	3	38
Sorgente et al. [9]	4	3	3	1	2	4	3	3	3	1	3	30
Chopra et al. [10]	4	4	4	3	3	4	4	4	3	2	4	39
Agnew et al. [11]	4	4	4	4	4	4	4	4	4	4	4	48
Montesi et al. [12]	3	3	3	1	3	4	3	3	3	2	3	31
Mancini et al. [14]	4	4	4	3	3	4	4	4	4	3	4	41
Aurich et al. [24]	3	3	3	1	2	4	3	3	3	1	3	29
van Dijk et al. [25]	4	4	3	1	3	4	4	3	3	2	4	35

The methodological quality of observational studies was evaluated using the NOS. This tool assesses quality based on three domains: selection of study groups (maximum 4 stars), comparability of the groups (maximum 2 stars), and outcome ascertainment (maximum 3 stars). A maximum of nine stars can be awarded, with higher scores indicating lower risk of bias and stronger methodological quality. The results of the quality assessments for each study are presented in Table 4.

**Table 4:** Quality assessment of observational studies using the NOS NOS domains include: Selection: Representativeness of the cohort, selection of the non-exposed cohort, ascertainment of exposure, and confirmation that the outcome was not present at the start; Comparability: Control for confounding factors; Outcome: Outcome assessment method, adequacy of follow-up duration, and completeness of follow-up. Scores are indicated by the number of stars (★); maximum total score = 9 NOS: Newcastle-Ottawa Scale

Study	Selection	Comparability	Outcome	Total (★/9)
Sawamoto et al. [1]	★★★★	★★	★★★	9
Lamiquiz‑Moneo et al. [13]	★★★★	★★	★★	8
González-Herrera et al. [21]	★★	★★	★★★	7
Coppedè et al. [23]	★★★	★	★★	6
Vitolo et al. [28]	★★★★	★★	★	7

The methodological quality of the included RCTs was evaluated using the CASP Randomised Controlled Trial Checklist. This tool comprises 11 structured questions designed to assess the validity, results, and applicability of clinical trial findings. Each trial was reviewed to determine whether it clearly defined its research question, used appropriate randomization, maintained group comparability, applied blinding where feasible, and fully reported treatment effects and outcomes. The results of the appraisal are summarized in the Table 5 for the three RCTs included in this review.

**Table 5:** Quality assessment of RCTs using the CASP checklist The CASP tool evaluates RCTs across three core areas: Validity (Questions 1–6): Assesses design aspects such as randomization, blinding, and treatment consistency; Results (Questions 7–8): Focuses on the size and precision of the treatment effect; Applicability (Questions 9–11): Examines whether results can be applied to practice and if benefits outweigh harms. Responses include “Yes,” “No,” or contextual explanations CASP: Critical Appraisal Skills Programme; RCT: Randomized controlled trial; CBT: Cognitive behavioral therapy

CASP Questions	Papandonatos et al. [5]	Hellberg et al. [20]	Palavras et al. [26]
1. Did the study address a clearly focused issue?	Yes	Yes	Yes
2. Was the assignment of participants to interventions randomized?	Yes	Yes	Yes
3. Were all participants who entered the study accounted for at its conclusion?	Yes	Yes	Yes
4. Were participants, staff, and study personnel "blind" to treatment?	No (open label)	No (open label)	No (CBT makes blinding impractical)
5. Were the groups similar at the start of the trial?	Yes	Yes	Yes
6. Aside from the experimental intervention, were the groups treated equally?	Yes	Yes	Yes
7. How large was the treatment effect?	Moderate genetic interaction	Statistically significant	Statistically significant CBT effect
8. How precise was the estimate of the treatment effect?	Confidence intervals reported	Confidence intervals reported	Confidence intervals reported
9. Can the results be applied to the local population?	With caution (North American cohort)	Yes	Yes (more general clinical context)
10. Were all clinically important outcomes considered?	Yes	Yes	Yes
11. Are the benefits worth the harms and costs?	Yes	Yes	Yes

Non-randomized intervention studies included in this review were evaluated using the ROBINS-I tool. This framework systematically assesses seven domains of potential bias: confounding, participant selection, intervention classification, deviations from intended interventions, missing data, measurement of outcomes, and selective reporting. Each domain is judged as low, moderate, serious, or critical risk of bias, leading to an overall judgment for each study. The purpose of this assessment is to determine the internal validity of the evidence generated from non-randomized study designs. The results of the ROBINS-I appraisal are presented in Table 6.

**Table 6:** Risk of bias assessment of non-randomized intervention studies using the ROBINS-I tool Each domain is rated as low, moderate, serious, or critical risk of bias. "Overall Risk of Bias" reflects the highest level of bias identified across domains. ROBINS: Risk of Bias in Non-randomized Studies of Interventions

Study	Bias Due to Confounding	Bias in Selection of Participants	Bias in Classification of Intervention	Bias Due to Deviations from Intended Interventions	Bias Due to Missing Data	Bias in Measurement of Outcomes	Bias in Selection of Reported Results	Overall Risk of Bias
Chen et al. [4]	Serious – no randomization, small sample, potential confounding factors	Moderate – voluntary sample; inclusion/exclusion reported	Low – same behavioral intervention for all	Moderate – adherence/fidelity not systematically tracked	Serious – handling of missing data unclear	Moderate – predictor/outcome measures may vary	Moderate – exploratory nature; possible selective reporting	Serious
Aller et al. [19]	Moderate – baseline differences may not be fully accounted for	Low – well-defined cohort; no randomization	Low – intervention consistently applied	Low – multidisciplinary advice likely adhered to	Moderate – missing outcome data not fully specified	Low – objective weight measures	Moderate – potential selective analysis	Moderate
de Luis et al. [22]	Serious – no randomization; confounders like lifestyle factors may bias results	Moderate – participant selection criteria unclear	Low – consistent intervention classification	Moderate – adherence to meal-replacement may vary	Serious – loss to follow-up and missing data not addressed	Moderate – some self-reported measures	Moderate – lack of pre-registration	Serious
Rigamonti et al. [23]	Serious – no comparator, potential confounding by age, socioeconomic status	Moderate – unclear selection methods	Low – program applied uniformly	Moderate – program fidelity not described	Serious – attrition and missing follow-up not reported	Serious – methylation and weight outcomes may be measured inconsistently	Moderate – potential multiple unreported analyses	Serious

Study Findings

Remarkably, individuals that identified high self-control and resilience in regulating food cues, cravings, and emotional eating were more likely to abide by long-term maintenance tenets [1]. Monogenetic traits were also always linked to variation in weight loss response, especially among participants on structured interventions on diet and lifestyle. The findings noted that the individuals carrying the AA genotype of the FTO locus had much higher risks of re-gaining weight, particularly during studies using diet only interventions or beyond 12 months. The AA allele was also specific contributing among those who had a of BMI of less than 35 [2]. Stratified analysis by country of Europe and studies with adjustment by baseline BMI also confirmed these findings. An observational cohort study shows the baseline BMI and parental support were significantly associated with weight loss success in adolescents. Children with higher levels of parental involvement had a greater mean weight reduction (mean ΔBMI z-score = -0.23, p < 0.05) [3]. The relevance of family-based behavioral therapy in pediatric obesity has been highlighted in the study. A pilot neuroimaging study links the activation in the prefrontal cortex and genetic markers to successful behavioral weight loss. Though sample sizes were small, participants showing greater neural activity in executive function areas had a statistically greater reduction in body weight (mean loss = 6.2%, p < 0.05), suggesting neurobiological predictors of intervention responsiveness [4].

Wider genetic risk constellations were also identified to affect weight trends, but not restricted to FTO. Examining the polygenic scores across participants in the Diabetes Prevention Program and Look AHEAD trials, it found that individuals with high genetic risk scores were significantly more likely to regain lost weight [5]. Their analysis revealed that even a modest 2% increase in body weight was positively correlated with higher genetic risk scores (p < 0.001). These results support the additive impact of genetic vulnerability to weight regulation and prove the feasibility of introducing polygenic risk scores when planning personalized interventions. Interestingly, individuals in the lowest genetic risk quartile (Q1) experienced the most pronounced reductions in body weight (-2.15 kg), whereas those in higher quartiles had less significant improvements, although no baseline weight differences were observed.

Systematic review of genetic predictors of weight loss after bariatric surgery also emphasized that several polymorphisms, notably in FTO, MC4R, and TMEM18 genes, have significant predictive value [6]. Successful maintainers consistently practiced high-frequency physical activity and self-weighing. Those who engaged in daily monitoring lost significantly more weight (mean = -13.6 kg vs -9.4 kg, p < 0.01), supporting behavioral adherence as a determinant of maintenance [7]. A systematic review demonstrated that psychological traits such as intrinsic motivation, emotional stability, and habit formation were stronger predictors of weight maintenance than demographic variables. For instance, participants with high intrinsic motivation had twice the odds (OR = 2.12, 95% CI: 1.45-3.10) of maintaining weight loss over 12 months [8].

Web-based interventions resulted in significant but small average weight reductions (~2.5 kg, p < 0.001). Programs with self-monitoring components had stronger effects (effect size d = 0.48) compared to passive education-only platforms [9]. The single-variable analysis points out behavioral and psychological differences between people who continued to lose weight and those whose results faded after 12 months. Some of the major factors linked to results were a decline in weight (ΔBW), less depression after the intervention (CES-D), greater binge eating (BES), more prohibited eating (disinhibition), and an increase in food addiction symptoms (YFAS) [10]. According to these results, keeping weight down is more common for people who have higher self-control and are mentally strong.

A Cochrane review found that lifestyle interventions in women with endometrial cancer were associated with improved cancer-specific survival and a potential reduction in cancer recurrence [11]. Greater than 5% loss in body weight was positively correlated with better progression-free survival [11]. Those combining behavioral therapy, physical activity, and diet led to the greatest long-term weight loss (mean loss maintained = -8.6 kg at 24 months, p < 0.001). The risk of attrition was considerably reduced in interventions using structured follow-up [12]. Another study demonstrated that specific genetic variants predict weight loss responsiveness among overweight and obese adults. The hypothesis that genotype has an effect on metabolic pathways and processes implicated in fat oxidation, appetite control and energy homeostasis hold water based on the fact that subjects with specific allelic patterns responded better to structured interventions [13].

The Mediterranean diet led to an average weight loss of 4.1 kg over 12 months (p < 0.001) and was superior to low-fat diets for long-term maintenance, particularly when olive oil and nuts were included [14]. Weight loss (as % of initial weight) during the 12 months of treatment for carriers of the combination of PPARγ rs1801282 C/G-GG and TIMP4 rs3755724 T/C, in comparison with carriers of all other genotype combinations of these PPARγ and TIMP4 single nucleotide polymorphisms (SNPs) [19].

Women with polycystic ovary syndrome (PCOS) who had expression changes in adipose genes related to insulin resistance experienced higher weight gain over time (mean BMI increase = 1.6 kg/m², p = 0.04). The expressions of adiponectin and leptin were particularly predictive [20]. Evidently, school-aged children with FTO risk alleles (rs9939609 A) had significantly higher obesity rates (OR = 2.45, p = 0.008), particularly in those with high-calorie diets and low physical activity [21]. PERILIPIN rs2289487 variant was associated with higher fat mass loss after a meal-replacement diet (-2.6 kg vs -1.4 kg, p = 0.02) in postmenopausal women, supporting its role as a genetic predictor of dietary response [22].

Identified DNA methylation changes in the CLOCK and BMAL1 genes that were associated with BMI reduction in obese adolescents after a 3-week intervention (mean BMI reduction = -1.4, p < 0.05). These epigenetic modifications indicate chronobiological responsiveness toward weight interventions [23]. Lifestyle-mediated weight loss induced DNA methylation changes in genes associated with adipogenesis and inflammation, suggesting that epigenetic plasticity may support sustained weight control. The research identified an average 1.9% increase in methylation changes in major functional areas following the intervention [24]. Evidently, studies on epigenetics and obesity show that concluded that early-life exposure to poor nutrition or sedentary behavior was associated with long-term DNA methylation changes in metabolic genes like LEP and POMC, contributing to higher BMI in adulthood [25].

A structured weight loss intervention delivered as a combination of novel psychotherapy with cognitive behavioral therapy (CBT) resulted in much better psychological and physical outcomes in metabolic responses compared to individuals with binge eating problems receiving a structured weight loss program alone. Over 16 weeks, participants gain a loss of weight by an average of 5.2 kg and a 57% decrease in binge episodes. They also demonstrated an increase in emotional regulation, eating restraint, self-efficacy, and decline in depressive symptoms [26].

A study analyzing DNA methylation pattern in appetite-regulating genes found that these epigenetic modifications could forecast weight loss following bariatric surgery [27]. Their finding suggests a possibility of differences in weight management-related gene expression that are environmentally and behaviorally induced. Another study supported this by showing that individuals heterozygous for the rs696217 SNP in the preproghrelin gene experienced more significant weight loss after surgery. Such observations underpin the validity of the existence of gene-environment interaction models that contribute to a lifestyle-inherited factors which is the key to a complete picture of the determinants of the outcomes of obesity treatment [28].

Discussion

Behavioral Traits as Consistent Predictors of Weight Loss Maintenance

The univariate and multivariate analyses found that several behavioral qualities tended to be present in maintained 12-month weight loss. Among all the variables, losing larger amounts of weight at the beginning (ΔBW) was the best indicator of who will keep the weight off over time. This opinion agrees with previous research, which suggests early weight management success can keep someone on track and motivate them to keep following a healthy lifestyle [20]. Lower disinhibition after the program and having fewer food addiction symptoms were also important factors linked to maintaining weight loss. A lower BES means people tended to have more control over what they eat, while better CES-D scores demonstrated the positive impact of good mental wellness on sticking to a healthy diet [21]. Also, it is interesting that before treatment, there was no significant difference in scores for these variables (CES-D, State-Trait Anxiety Inventory (STAI), BES) between those who finished and those who did not. It shows that post-treatment changes are more significant indicators of how well a treatment worked, rather than the initial state of the patient [22]. As a result, flexibility in behavior matters more than fixed personality traits for long-term results.

Genetic Influences: FTO, Genetic Scores, and SNP Combinations

Several studies have stressed that genetic variations in FTO and PPARγ, and combinations with TIMP4, greatly affect these diseases. From the findings, those with the AA variant of FTO lost weight but regained it more often, especially among people with a normal BMI, who were on diets, and who participated in research for a longer period. It is notable that TA variants had less of an effect, meaning that AA carried the stronger risk [23]. These results revealed that those with higher total genetic risk scores tended to experience worse results. Individuals who gained more than 5% body-weight scored much higher than those who stayed the same or lost weight [24]. Individuals categorized as having the lowest genetic risk experienced the biggest increase in weight loss; hence, polygenic scores are reliable. Additionally, the carriers of PPARγ rs1801282 C/G-GG and TIMP4 rs3755724 T/C genotypes responded more favorably to multidisciplinary interventions, suggesting a gene-treatment interaction. These results align with studies that propose gene variants can influence metabolism, appetite, and response to dietary interventions [25].

Gene × Behavior Interactions: A Precision Approach

Perhaps the most critical insight from this review is the interaction between genetic predisposition and behavioral effort. While certain genotypes (e.g., FTO AA) predispose individuals to regain weight, this risk is not deterministic. For example, participants with high-risk genotypes but who adhered to structured dietary interventions experienced less weight regain than those in less structured or mixed programs. This suggests that behavioral interventions can moderate genetic risk. Thus, this gene × behavior interaction aligns with the principles of precision health, where genetic insights are used to tailor behavioral strategies. For instance, individuals with high disinhibition or food addiction scores, combined with FTO risk alleles, might benefit from CBT integrated into weight programs [26]. Likewise, those with favorable genotypes could be encouraged to engage in more autonomous self-regulation strategies [27]. Moreover, the regression demonstrated that behavioral variables (e.g., post-disinhibition, YFAS) retained their predictive value even after adjusting for weight loss, further emphasizing the independent and modifiable nature of these factors [28].

Role of Family Support and Socioeconomic Factors in Long-Term Weight Loss Maintenance

A general perception about long-term weight loss maintenance is that it is nearly impossible to succeed [29]. Weight loss can be achieved through various means and modalities, but long-term maintenance of lost weight is much more challenging. Therefore, treatment of obesity requires continuous clinical attention and targeted counseling to support sustainable healthful behaviors and positive weight regulation to achieve weight maintenance [30]. Hall and Kahan, in their study, have suggested a collective role, where everybody in the field of care and patients themselves need to understand that, since obesity is a chronic metabolic condition, weight management will need to be lifelong [31]. Further, in their topic analysis, Phelan et al. reveal that factors that prompted successful weight loss among participants included, among others, “Social Prompts,” which described the role other people (friend, doctor, husband, daughter, child) played in prompting their weight loss [32]. These same supports, together with group and societal support, were instrumental in keeping participants more accountable in maintaining their weight loss [32]. Other factors include time and money involved in funding and maintaining healthful behaviors and diets, as several people join and rejoin the weight loss journey several times due to a lack of time and/ or money [32]. Positive support, like giving compliments and active participation of friends and family, appears beneficial in weight loss maintenance [33].

Limitations of the study

Despite offering valuable insights, the reviewed literature revealed several gaps and limitations that hinder broader applicability and generalization. A key issue is the lack of standardization in defining "long-term" weight loss maintenance. While some studies used a ≥ 5% body weight change as the threshold, others used ≥ 10%, with follow-up durations ranging from six months to two years. These inconsistencies complicate data comparison and reduce the precision of meta-analytic interpretations. Standardizing outcome definitions is, therefore, critical for future research. Most studies focused on North America and Europe, with very few being done in areas such as Africa and Latin America. Such an understanding does not take into account the major contributions of cultural, economic, and environmental components to weight management and obesity. Furthermore, studies often ignored gender-based analyses, even though research brought to light that men and women do not always react alike to different interventions. There is also a large gap due to the lack of models that link genetics and behavior together. A lot of these studies examined these domains apart, which does not fully take advantage of whole-person care. Generally, family support, access to nutritious food, and socioeconomic background were not usually considered as key factors. In addition, studies using small groups of subjects weakened their statistical strength. More collaborative work conducted across different sites is necessary to improve research on the connection between genes and behavior. Lastly, the follow-up duration is another notable limitation, as it is shorter (two years). Thus, longer-term study follow-up durations (more than two years) are needed as they are likely to reveal additional behavioral and physiological factors.

## Conclusions

In conclusion, long-term weight loss maintenance is influenced by both behavioral and genetic factors. High physical activity, dietary restraint, and improved psychological health support success, while FTO variants and high genetic risk scores increase relapse risk. However, structured behavioral interventions can reduce genetic risk. Personalized approaches combining genetics and behavior may improve long-term outcomes in obesity management.
